# Regulating Subcellular Metal Homeostasis: The Key to Crop Improvement

**DOI:** 10.3389/fpls.2016.01192

**Published:** 2016-08-05

**Authors:** Khurram Bashir, Sultana Rasheed, Takanori Kobayashi, Motoaki Seki, Naoko K. Nishizawa

**Affiliations:** ^1^Plant Genomics Network Research Team, Center for Sustainable Resource Science, RIKEN, Yokohama Campus, YokohamaJapan; ^2^Kihara Institute for Biological Research, Yokohama City University, YokohamaJapan; ^3^Research Institute for Bioresources and Biotechnology, Ishikawa Prefectural University, NonoichiJapan; ^4^Core Research for Evolutional Science and Technology – Japan Science and Technology Agency, KawaguchiJapan; ^5^Graduate School of Agricultural and Life Sciences, The University of Tokyo, TokyoJapan

**Keywords:** biofortification, chloroplast, iron, manganese, metabolome, metal transport, mitochondria, zinc

## Abstract

Iron (Fe), zinc (Zn), manganese (Mn), and copper (Cu) are essential micronutrient mineral elements for living organisms, as they regulate essential cellular processes, such as chlorophyll synthesis and photosynthesis (Fe, Cu, and Mn), respiration (Fe and Cu), and transcription (Zn). The storage and distribution of these minerals in various cellular organelles is strictly regulated to ensure optimal metabolic rates. Alteration of the balance in uptake, distribution, and/or storage of these minerals severely impairs cellular metabolism and significantly affects plant growth and development. Thus, any change in the metal profile of a cellular compartment significantly affects metabolism. Different subcellular compartments are suggested to be linked through complex retrograde signaling networks to regulate cellular metal homeostasis. Various genes regulating cellular and subcellular metal distribution have been identified and characterized. Understanding the role of these transporters is extremely important to elaborate the signaling between various subcellular compartments. Moreover, modulation of the proteins involved in cellular metal homeostasis may help in the regulation of metabolism, adaptability to a diverse range of environmental conditions, and biofortification. Here, we review progress in the understanding of different subcellular metal transport components in plants and discuss the prospects of regulating cellular metabolism and strategies to develop biofortified crop plants.

## Introduction

Metals, such as iron (Fe), zinc (Zn), copper (Cu), and manganese (Mn), are essential for all higher organisms (Marschner, 1995). Fe readily changes its oxidative state, which allows participation in cellular functions, and its regulation is extremely important to avoid cellular toxicity. Fe participates in cellular respiration, synthesis, and stabilization of chlorophyll, photosynthetic electron transport, and various other metabolic functions (Grotz and Guerinot, 2006). The most obvious symptom of Fe-deficiency in plants is chlorosis due to a decrease in chlorophyll content, which significantly affects plant growth, development and product quality. Cu is also a cofactor for various enzymes involved in respiration and photosynthesis and is toxic at higher concentrations. In plants, Zn does not readily change its oxidative state and does not take part in oxidoreduction reactions (Marschner, 1995). Zn is integral to enzymes involved in protein, nucleic acid, carbohydrate, and lipid metabolism (Ishimaru et al., 2011; Suzuki et al., 2012). Zn is also critical for proteins containing DNA-binding Zn-finger motifs, RING fingers, LIM domains, proteins associated with DNA and RNA synthesis, such as transcription factors, RNA polymerases, and reverse transcriptase (Broadley et al., 2007; Bashir et al., 2012). Mn is a cofactor or activator of enzymes, such as oxalate oxidase, Mn superoxide dismutase, RNA polymerase, malic enzyme, isocitrate dehydrogenase, and phosphoenolpyruvate carboxykinase, and it is required for photosynthetic oxygen evolution in chloroplasts (Marschner, 1995; Socha and Guerinot, 2014).

Various cellular organelles, such as chloroplasts and mitochondria, depend on Fe, Cu, Mn, and Zn for their activities, and deficiencies and toxicity of these minerals significantly disturb chloroplast and mitochondrial functioning, which ultimately hinders plant growth and development (Bashir et al., 2011a,b; Duy et al., 2011; Vigani et al., 2013b). Thus, regulating the uptake and cellular distribution of these minerals is extremely important for optimal cellular functioning and could lead to breeding crop plants with better adaptability to changing environments and may contribute to providing healthy food with improved mineral contents. Regulation of Fe uptake from the rhizosphere has been reviewed extensively (Kobayashi and Nishizawa, 2012). Genes involved in Fe acquisition in plants are induced under Fe-deficient conditions, and various genes putatively acting as Fe sensors have been discussed (Kobayashi and Nishizawa, 2014). Uptake of Zn, Cu, and Mn is also reasonably well understood and has been reviewed (Palmer and Guerinot, 2009; Yruela, 2009; Bashir et al., 2012; Jain et al., 2014; Socha and Guerinot, 2014). Here, we review the proteins controlling mineral distribution in various cellular organelles and discuss the prospects of regulating these proteins for optimized metabolism and biofortification purposes.

## Cellular Metal Homeostasis

**FIGURE 1:**
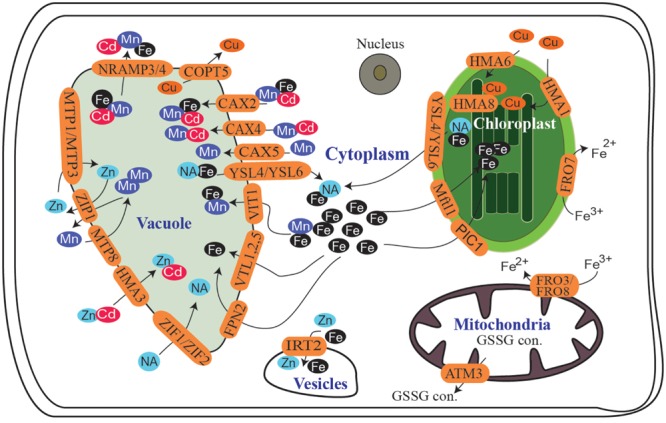
**Summary of subcellular metal transport in *Arabidopsis.*** Proteins participating in Fe, Mn, Cu, or Zn transport into or out of different cellular organelles are shown. NA, nicotianamine; GSSG con., glutathione conjugates.

Plants absorb metals from the soil for transport to the cytoplasm and specific subcellular compartments, such as the nucleus, mitochondria, and chloroplasts, where they perform specific functions, whereas vacuoles serve as a reservoir to regulate cell metallic balance. Cellular metal transport is governed by a diverse set of membrane proteins. As free metals are toxic, plants utilize various chelating agents, such as nicotianamine (NA), deoxymugineic acid (DMA), citrate, ascorbate, and phenolics, to solubilize these metals and protect cells from oxidative damage (Jeong and Guerinot, 2009; Palmer and Guerinot, 2009; Bashir et al., 2013a; Grillet et al., 2014). Transporters belonging to various families actively participate in the regulation of cellular metal homeostasis (Vigani et al., 2013a; Boutigny et al., 2014; Jain et al., 2014; Socha and Guerinot, 2014; Finazzi et al., 2015; López-Millán et al., 2016). Various members of the Yellow stripe-like (YSL) family (named for the phenotype of mutants from which the maize Yellow Stripe 1 was cloned), zinc-regulated transporter/iron-regulated transporter (ZRT/IRT)-related protein (ZIP) family, natural resistance associated macrophage protein (NRAMP) family, cation diffusion facilitator (CDF) family, major facilitator super family (MFS), P_1B_-type heavy metal ATPase (HMA) family, the vacuolar iron transporter (VIT) family, and the cation exchange (CAX) family play significant roles in cellular metal homeostasis (**Figure 1**). Understanding the function and regulation of these transporters is extremely important to characterize the factors governing micronutrient uptake and distribution in plants.

## Chloroplast Metal Homeostasis

Metals play an extremely important role in chlorophyll synthesis, stabilization and photosynthesis; thus, regulating metal homeostasis in chloroplasts is extremely important (Finazzi et al., 2015; López-Millán et al., 2016). In plants, the Fe storage protein ferritin localizes mainly to chloroplasts; thus, chloroplasts serve as a storage hub for Fe, particularly during early plant growth (Briat et al., 2010). The roles of Fe, Mn, and Cu in photosynthesis and metabolism have been discussed extensively (Nouet et al., 2011; Vigani et al., 2013a; López-Millán et al., 2016), so we will not discuss these details here. The *Arabidopsis* ferric chelate reductase oxidase 7 (FRO7), which reduces ferric ion to Fe^2+^, localizes to chloroplasts and plays a significant role in Fe homeostasis (Jeong et al., 2008; Jain et al., 2014). Permease in chloroplasts 1 (PIC1) is important for Fe transport to chloroplasts (Duy et al., 2011), and the role of *Arabidopsis* mitoferrin-like 1 (AtMfl1) in Fe transport to chloroplasts has also been discussed, although there is no direct evidence that AtMfl1 transports Fe (Tarantino et al., 2011). An increase or decrease in *PIC1* expression significantly alters chloroplast development and plant growth through reactions triggered by Fe toxicity or Fe-deficiency. These results indicate that reciprocal signaling between chloroplasts and nuclear genes may exist (Duy et al., 2011; López-Millán et al., 2016). Contrasting reports on the localization and function of AtYSL4 and AtYSL6 have been published (Conte et al., 2013; Divol et al., 2013). According to Divol et al. (2013), *Arabidopsis* YSL4 and YSL6 localize to chloroplasts and play a major role in releasing chloroplastic Fe-NA, with a particularly important role during embryogenesis and senescence.

Importation of Cu into chloroplasts is mediated mainly by a chloroplast-envelope P_IB_-type ATPase, called HMA6 (also known as PAA1), which transports Cu^+^ and is required for photosynthesis (Catty et al., 2011; Boutigny et al., 2014). Copper may also be imported by HMA1, which transports Cu^2+^ (Boutigny et al., 2014). HMA1 and HMA6 behave as distinct pathways for importing Cu (Boutigny et al., 2014). The *HMA1* and *HMA6* double mutants do not exhibit a lethal phenotype, suggesting that Cu could enter the chloroplast through another, yet unknown, transporter (Catty et al., 2011; Boutigny et al., 2014). The role of HMA8 (PAA2) in Cu transport to chloroplast thylakoid membrane has also been documented (Aguirre and Pilon, 2016).

Despite the essential role of Mn in photosynthesis, Mn transporters localizing to the chloroplast membrane have not been reported. Similarly, although HMA1 is suggested to be involved in the control of Zn homeostasis in chloroplasts (Williams and Mills, 2005; Kim et al., 2009), its role in Zn transport is controversial (Boutigny et al., 2014), and the protein(s) transporting Zn to and from chloroplasts have not been clearly documented. Characterization of chloroplast transporters would provide a way to regulate metal homeostasis in chloroplasts and ultimately improve crops. Regulating metal transport to and from the chloroplast could be utilized to improve photosynthesis, improve cellular metabolism, and develop crop plants with a better ability to grow under changing environmental conditions.

## The Vacuole Is a Major Reservoir for Metal Homeostasis

The vacuole serves as a major reservoir to regulate cellular metal homeostasis. To avoid metal toxicity, excess metals are deposited in vacuoles for withdrawal when required. In plants, several efflux and influx transporters localized to the vacuolar membrane have been reported. In *Arabidopsis*, FPN2 (also known as IREG2) deposits cytoplasmic Fe in vacuoles, whereas vacuolar Fe transporter 1 (VIT1) deposits Fe and Mn in vacuoles (Kim et al., 2006; Schaaf et al., 2006; Morrissey et al., 2009). Changes in *VIT1* expression significantly alters Fe localization in *Arabidopsis* seeds (Kim et al., 2006). *Arabidopsis* VIT1-like (VTL) proteins have also been characterized (Gollhofer et al., 2014). *Arabidopsis* VTL1, VTL2, and VTL5 are functional Fe transporters that play a role in transferring cytoplasmic Fe into vacuoles and contribute to Fe homeostasis regulation *in planta* (Gollhofer et al., 2014). It is unclear why *Arabidopsis* requires so many vacuolar Fe transporters and whether VTL proteins also transport other metals. In rice, VIT1 and VIT2 are functional vacuolar transporters that contribute to Fe, Mn, and Zn homeostasis. Disrupting VIT1 or VIT2 function leads to increased metal accumulation in rice seeds (Zhang et al., 2012). Disrupting VIT also alters petal colors in flowers, due to changes in the accumulation of pigments that complex with metals (Momonoi et al., 2009; Yoshida and Negishi, 2013).

Natural resistance associated macrophage proteins are integral membrane proteins involved in transport of Mn, Fe, and Cd. The mobilization of Fe (as well as Mn and Cd) from vacuoles in *Arabidopsis* is mediated by NRAMP3 and NRAMP4. *Arabidopsis* NRAMP3 and NRAMP4 release Fe from vacuoles under limited Fe conditions or when demand increases (Lanquar et al., 2005; Mary et al., 2015; Pottier et al., 2015). AtNRAMP3 and AtNRAMP4 function oppositely to AtVIT1. One study revealed that preventing Fe storage in endodermal vacuoles through AtVIT1 knockout rescues the Fe mobilization defect in the *nramp3nramp4* double mutant under optimal conditions (Mary et al., 2015).

The role of *Arabidopsis* YSL4 and YSL6 in remobilizing vacuolar Fe has also been discussed (Conte et al., 2013). In contrast to Conte et al. (2013), Divol et al. (2013) reported that AtYSL4 and AtYSL6 localize to vacuolar and internal membranes resembling endoplasmic reticulum (ER) and were suggested to play a role in heavy metal stress responses. *AtYSL4* and *AtYSL6* knockout mutants grow better under excess Mn and exhibit improved growth of roots exposed to excess Mn and excess nickel (Ni), whereas overexpression of *YSL6* results in decreased root growth under excess Mn or Ni. In this context, it is reasonable to believe that AtYSL4 and AtYSL6 may help provide Mn and Ni-NA complexes to proteins located in internal cellular compartments (Conte et al., 2013).

Metal tolerant protein 1 (MTP1) and MTP3 in *Arabidopsis* are critical for sequestering excess Zn into the vacuole (Desbrosses-Fonrouge et al., 2005; Arrivault et al., 2006). Zn deposition in the vacuole is also mediated by HMA3, which sequesters Cd (Morel et al., 2009). *Arabidopsis* ZIP1 is a vacuolar Zn and Mn transporter and may play a role in remobilizing Mn and/or Zn from the vacuole to the cytoplasm in roots (Milner et al., 2013). Efflux of NA 1 (ENA1) is a member of the MFS family that transports NA to the vacuole in rice (Nozoye et al., 2011). ENA1 homologs, Zn-induced facilitator 1 (ZIF1) and ZIF2, have been characterized in *Arabidopsis* (Haydon and Cobbett, 2007; Haydon et al., 2012; Remy et al., 2014). *Arabidopsis* ZIF1 and ZIF2 localize to the vacuole and may play a role in tolerance to excess Zn (Haydon and Cobbett, 2007; Haydon et al., 2012; Remy et al., 2014). The ZIF1 could also play important role in Fe transport under Fe deficient conditions (Haydon et al., 2012). The role of ZIFL2 to mediate potassium and cesium influx in *Arabidopsis* has also been reported recently (Remy et al., 2015).

*Arabidopsis* CAX2 transports Fe, Cd, and Mn; CAX4 transports Mn and Cd, and CAX5 transports Mn to the vacuole (Socha and Guerinot, 2014). The role of VIT1 in sequestering Mn to the vacuole has also been discussed. *Arabidopsis* MTP8 belongs to the CDF family of transporters and was identified by screening T-DNA mutants on high pH medium with decreased Fe availability (Eroglu et al., 2016). MTP8 is a vacuolar Mn transporter, which sequesters excess Mn to the vacuole, and plays a major role protecting plants from Mn toxicity. *MTP8* expression increases under low Fe conditions as well as under high Mn conditions (Eroglu et al., 2016). *mtp8* mutants are more sensitive to Fe-deficiency in the presence of Mn due to the decrease in ferric chelate reductase activity (Eroglu et al., 2016). Alternately this increased sensitivity could also result from an inability to store the Mn taken up by ZIP family Fe regulated transporter 1 (IRT1). IRT1, is involved in Cd, Fe, Mn, and Zn transport into the cell. Unlike other plants, rice accumulates more Mn in shoots compared with roots (Ishimaru et al., 2012), and rice MTP8.1 seems to be the major gene sequestering excess Mn into the vacuole (Chen et al., 2013). OsVIT1 and OsVIT2 may also contribute to sequestration of Mn into the vacuole (Zhang et al., 2012). Vacuolar Mn remobilization in *Arabidopsis* is controlled by NRAMP3, NRAMP4, and ZIP1. The *Arabidopsis* Cu transporter (COPT/Ctr) family members COPT1, COPT2, COPT3, and COPT5 have been implicated in Cu transport (Grotz and Guerinot, 2006). Among these, COPT5 pumps vacuolar Cu into the cytoplasm and plays an integral role in photosynthetic electron transport in chloroplasts (Garcia-Molina et al., 2011; Klaumann et al., 2011).

Mineral transporters also interact with toxic metals as described above. For example, HMAs are members of a diverse family of transporters localized in the plasma membrane as well as subcellular compartments, and they are involved in translocation or detoxification of Cu, Zn, and Cd. *Arabidopsis* HMA3 sequesters Cd and Zn, (may also be involved in Co, and Pb sequestration) to the vacuole, whereas OsHMA3 seems very specific to Cd sequestration (Morel et al., 2009; Ueno et al., 2010; Miyadate et al., 2011). In *Arabidopsis*, CAX2, CAX4. NRAMP3 and NRAMP4 also transport Cd. The expression of vacuolar transporters may or may not be regulated by the cytoplasmic status of the plants; e.g., *ZIF1* (Haydon and Cobbett, 2007), *OsVIT2* (Zhang et al., 2012), *CAX4* (Mei et al., 2009), which regulate metal sequestration into vacuoles, are upregulated under excess metal conditions. *MTP3* expression is positively regulated by Fe deficiency and by excess Zn and Co (Arrivault et al., 2006). On the other hand, *Arabidopsis VIT1* expression (Kim et al., 2006; Zhang et al., 2012), *MTP1* (Kobae et al., 2004; Kawachi et al., 2009) and *COPT5* (Garcia-Molina et al., 2011; Klaumann et al., 2011) remain unaffected by metal status (Peng and Gong, 2014). It is important to mention that basic helix-loop-helix protein FER-like Fe deficiency-Induced Transcription factor (FIT) regulates the expression of various genes (such as *IREG2*, *MTP3*, *MTP8*) to cope with metal excess resulting from increased expression of *IRT1* under Fe deficiency (Schmidt and Buckhout, 2011).

## Mitochondrial Metal Homeostasis

Mitochondria have evolved through endosymbiosis and retain numerous functions, such as synthesis of heme and lipoic acid cofactors, as well as assembly of bacterial Fe–S cluster (ISC) machinery (Lill et al., 2015). The ISC components are essential for other cellular compartments, as assembly of cytosolic and nuclear Fe/S proteins depends on the generation of mitochondrial S-containing compounds (Lill et al., 2015). *Arabidopsis* ATM3 (also known as STA1) exports glutathione trisulfide complexes from mitochondria to the cytoplasm (Schaedler et al., 2014), and defects in transport significantly affect Fe–S cluster assembly and molybdenum cofactor assembly in the cytosol (Kushnir et al., 2001; Bernard et al., 2009; Teschner et al., 2010), leaving plants chlorotic. In addition to its role in Fe–S cluster assembly, glutathione plays an important role in the plant’s response to metal availability (Bashir et al., 2007; Shanmugam et al., 2015). Glutathione plays an essential role in Fe-deficiency tolerance and NO-mediated Fe-deficiency signaling in *Arabidopsis* (Shanmugam et al., 2015).

Based on the vital functions of mitochondria in plants, it seems reasonable to conclude that mitochondria and/or other cellular organelles may regulate retrograde signaling pathways to control Fe deficiency-induced responses; however, the nature of the pathway is not clear (Vigani et al., 2013a,b). Plant mitochondria serve as a central hub of redox regulation and energy conversion by linking metabolic pathways from different subcellular compartments. These properties make mitochondria an ideal sensor to reflect cellular energetic and metabolic statuses (Sweetlove et al., 2007; Millar et al., 2011). As mitochondria serve as the powerhouse of the cell, changes in cellular energy status can reconfigure mitochondrial activities. As a result, changes in mitochondrial status could affect functions in other cellular compartments, such as changes in photosynthetic activity or nuclear gene expression (Schwarzländer et al., 2012; Vigani et al., 2013b).

Transport of metals into plant mitochondria is poorly understood, as only a few proteins involved in metal transport to and from mitochondria have been characterized. *Arabidopsis* FRO3 and FRO8 are mitochondrial proteins that putatively participate in ferric reduction in the mitochondrial membrane and contribute to mitochondrial Fe transport (Jain et al., 2014). The protein that transports cytoplasmic Fe into mitochondria has been characterized in rice. The rice mitochondrial Fe transporter (MIT) is essential for growth and development, as the rice *MIT* knockout mutant (*mit-1*) is lethal, and the *MIT* knockdown mutant (*mit-2*) exhibits reduced chlorophyll content and significantly affected plant growth. *mit-2* plants accumulate less Fe in mitochondria and significantly higher Fe in leaves and show symptoms of Fe deficiency, i.e., reduced chlorophyll content and upregulation of genes normally induced by Fe deficiency (Bashir et al., 2011a,b). Partial loss of MIT function in rice results in altered respiration and decreased total and mitochondrial aconitase activities, which possibly affect synthesis of the Fe–S cluster at the mitochondrial and cytosolic levels (Bashir et al., 2011b; Vigani et al., 2016). The yeast mitochondrial Fe transporter MRS3 also contributes to Cu transport (Vest et al., 2016). It is unclear if MIT contributes to transport of other divalent cations, particularly Cu. Mn accumulation in leaves as well as Mn and Cu accumulation in isolated mitochondria of *mit-2* plants is significantly altered (Bashir et al., 2011b). These changes could be due to either upregulation of other metal transporters, such as *OsVIT2*, or a direct effect of reduced MIT activity. The transporters involved in mitochondrial Mn, Cu, or Zn homeostasis have not been reported in plants. Two homologs of MIT have been identified in *Arabidopsis (*Jain, 2014*)*.

Besides these transporter proteins, mitochondrial ferritin and frataxin also play a role in mitochondrial metal homeostasis. Fer4 in *Arabidopsis* localizes to mitochondria (Murgia and Vigani, 2015). Defects in *Arabidopsis* frataxin result in an embryonic lethal phenotype; thus, a *frataxin* knockout mutant can only be maintained as a heterozygote (Murgia and Vigani, 2015). Mitochondrial respiration is not affected in ferritin- or frataxin-impaired *Arabidopsis*; however, these disruptions significantly alter accumulation of several minerals in *Arabidopsis* leaves. The mitochondrial iron regulated (MIR) protein in rice plays an undefined role in metal homeostasis. *MIR* expression is significantly upregulated in response to Fe deficiency, and the expression of metal homeostasis-related genes and accumulation of Fe changes significantly in *MIR* knockout mutants (Ishimaru et al., 2009). The MIR seems very specific to rice, as its homologs have not been identified in other crop species (Ishimaru et al., 2009).

## Vesicles and the Golgi Complex Play a Significant Role in Cellular Metal Homeostasis

Metal distribution in the nucleus, Golgi complex, and vesicles is important for cellular function (Roschzttardtz et al., 2011; Seo et al., 2012), and various proteins localized to these compartments have been described. The nucleus contains a significant amount of Fe (Roschzttardtz et al., 2011); however, it is not clear if transporters contributing to Fe transport (and other metals) into the nucleus exist in plants. The pore size of nucleus is large enough to allow metal movement, however, the excess metals may be extremely toxic for the nucleus thus there is possibility that nucleus may be regulating the metal movement. *Arabidopsis* IRT2 localizes to vesicles within root epidermal cells (Conte and Walker, 2011). AtIRT2 transports Fe^2+^ and is regulated by Fe deficiency. *Arabidopsis* MTP11 plays a role in Mn transport and tolerance by sequestering Mn into internal organelles (Delhaize et al., 2007).

Rice YSL6 knockout mutants are specifically sensitive to high Mn concentrations, and OsYSL6 is suggested to be involved in detoxifying excess Mn in rice; however, its cellular localization is not clear (Sasaki et al., 2011). Barley YSL5 localizes to vesicle membranes and is suggested to play a role in transport of Fe and/or mugineic acids (Zheng et al., 2011). *HvYSL5* expression is specifically regulated by Fe and not by Cu, Zn, or Mn. It is also possible that OsYSL5 and/or OsYSL6 function in transporting the metal-NA or metal-DMA complex into internal compartments. HvYSL5, OsYSL5, and OsYSL6 formed a distinct clade with AtYSL4 and AtYSL6 in a phylogenetic analysis; thus, these transporters may also play a role in subcellular metal homeostasis in barley (HvYSL5) and rice (OsYSL5 and OsYSL6; Aoyama et al., 2009; Zheng et al., 2011). Interestingly, proteins encoded by NAS genes localize to vesicles in rice but not in *Arabidopsis* (Nozoye et al., 2014a,b); thus, it would be very interesting to determine if the roles of OsYSL5 and OsYSL6 are different from those of *Arabidopsis* YSL4 and YSL6. Metal transporters have also been identified in the endomembrane system, transporting metals to the ER and/or Golgi. Golgi-localized barley MTP8.1 and MTP8.2 are Mn efflux transporters (Pedas et al., 2014). *MTP8.1* expression increases under Mn deficiency and toxicity in barley roots, whereas expression of *MTP8.2* decreases. *MTP8.1* and *MTP8.2* expression in leaves decreases in response to excess Mn. These proteins are suggested to play a significant role in Mn loading into the Golgi apparatus and in delivering Mn via secretory vesicles (Pedas et al., 2014). *Arabidopsis* HMA7 (also called RAN1) transports Cu for synthesis of ethylene receptors (Binder et al., 2010), and the IAR1 may be involved in Zn transport to ER (Lasswell et al., 2000).

## Regulating Metabolism by Manipulating Metal Homeostasis

Metals compete in that increased availability of one metal significantly alters transport of other metals (Bashir et al., 2014; Vigani et al., 2016). The cellular response to metal accumulation is very complex and employs different signaling mechanisms. These responses can be triggered by transporters moving more than one metal or due to increased competition among metals. Changes in the accumulation of metals in different subcellular compartments significantly affect the genes involved in metal transport and are also suggested to affect metabolism (Vigani et al., 2013a, 2016; Bashir et al., 2014). The metal requirement of chloroplasts is particularly high. Photosynthesis is the major activity of chloroplasts and a site of primary and secondary metabolism; thus, it is not surprising that changes in the accumulation of Fe or other metals alter chloroplast activities and, in turn, nuclear gene expression and cellular metabolism. Mutants and *PIC1*-overexpressing plants significantly alter chloroplast development and plant growth through Fe toxicity or Fe-deficiency responses (Duy et al., 2011; López-Millán et al., 2016), indicating the possibility of reciprocal signaling between chloroplasts and nuclear genes (Duy et al., 2011; López-Millán et al., 2016). Thus regulating the expression of *PIC1* through a stress inducible promoter could be an important strategy to regulate the optimal cellular metabolism and ultimately plant growth and development.

Transcriptomic and metabolomic profiling of *mit-2* reveals that retrograde signaling between the nucleus and mitochondria significantly modulates cellular gene expression and metabolism (Vigani et al., 2016). Interestingly, these changes are differentially regulated in roots and shoots, highlighting the role of mitochondria in response to the different energy needs of these tissues. In general, more significant changes are observed in the shoot transcriptome and metabolome of *mit-2* plants compared with those in root tissue. For example, oligosaccharides in the raffinose family along with pyruvic acid, fumaric acid, and ornithine specifically accumulate in shoot tissue (Vigani et al., 2016). The rice *OsOPT7* mutant *(opt7-1*) accumulates twofold more Fe in shoots compared with that in wild-type plants (Bashir et al., 2011b, 2015). This phenotype significantly resembles *mit-2* phenotype, however, the transcriptomic changes in *opt7-1* are significantly different from those in *mit-2*, mainly because Fe content and functionality are significantly altered in *mit-*2 mitochondria. However, some changes may be attributed to differential storage of Fe. Additional Fe is probably stored in vacuoles in the *mit-2* mutant versus in chloroplasts in the *osopt7-1* mutant (Bashir et al., 2011b, 2015; Vigani et al., 2016). Moderately increased availability of metals seems to trigger metabolism (Bashir et al., 2014), thus carefully regulating metal transporters to deliver metal supply to different subcellular compartments may be a way to improve metabolism and ultimately crop yield and product quality.

It has already been reported that changes in vacuolar and mitochondrial Fe transporters affect metal localization in *Arabidopsis* and rice seeds (Kim et al., 2006; Zhang et al., 2012; Bashir et al., 2013b; Mary et al., 2015). Thus, carefully regulating the expression of subcellular metal transporters may help to biofortify food crops with essential micronutrients. Due to the complex nature of substrate selection by different transporters, increased expression and activity of transporters such as *IRT1* result in excessive uptake of Mn, Ni, and Zn, as well as some other heavy metals. Under such circumstances, vacuolar sequestration of excess heavy metals is extremely important (Eide et al., 1996; Korshunova et al., 1999; Baxter et al., 2008). Regulating substrate-specific vacuolar metal transporters, such as NRAMP3 and NRAMP4, can also eliminate toxic metals (such as Cd) from the food chain (Pottier et al., 2015). This could be achieved by developing plants that efficiently deposit toxic metals into root and/or shoot vacuoles, efficiently remobilizing the essential metals, while lacking the ability to remobilize toxic metals from the vacuole. HMA3 effectively performs this function in rice, contributing to significantly reduced toxic Cd in rice grains (Ueno et al., 2010; Miyadate et al., 2011; Sasaki et al., 2014). Changes in the regulation of genes involved in metal accumulation and/or homeostasis are also observed in plants exposed to different biotic and abiotic stressors (Expert et al., 2012; Lei et al., 2014; Rasheed et al., 2016), highlighting the importance of metal distribution to mitigate the harmful effects of these stressors. Thus, regulation of subcellular metal distribution/homeostasis may help plants adapt to diverse environmental conditions and exhibit tolerance to various biotic and abiotic stressors.

## Conclusion

Regulation of cellular and subcellular metal homeostasis is extremely important for maintaining optimized metabolic rates and cellular functioning. The components of Fe homeostasis are understood reasonably well; however, the proteins involved in subcellular Zn and Mn and, to some extent, Cu homeostasis are poorly understood. Mutations in genes involved in metal uptake into the cell from the apoplasm often result in disturbed growth; however, mutations in genes involved in vacuolar metal homeostasis (e.g., *VIT* and *NRAMPs*) may positively affect growth and thus help in biofortification of crop plants. Fe, Zn, Mn, and Cu are essential for a variety of metabolic reactions. Metabolome significantly changes under various abiotic stresses. Thus regulating the distribution of micronutrients to various subcellular compartments could be useful strategy to regulate metabolome under adverse climatic conditions. This could be achieved by using promoters, differentially regulating the expression of genes involved in subcellular metal transport under different abiotic stresses. In particular, maintaining the micronutrient balance between chloroplast, mitochondria and cytoplasm could significantly improve the metabolism and plant growth, particularly under stress conditions. Metabolic profiling of the *mit-2* mutant demonstrated that subcellular metal distribution is important for regulating cellular metabolism, whereas *osvit1* and *osvit2* mutants clearly support the use of these proteins for biofortifying crop plants with essential micronutrients (Bashir et al., 2011b, 2013b; Zhang et al., 2012; Vigani et al., 2016). Although overexpressing *AtVIT1* in cassava resulted in increased Fe accumulation demonstrating that AtVIT1 could significantly contribute to Fe biofortification (Narayanan et al., 2015). However, it should be noted that over accumulation of Fe in vacuole may leave the cytoplasm Fe deficient and ultimately disturbing the cell metabolism and plant growth. Thus, it would be reasonable to conclude that manipulating the mineral distribution in a particular subcellular compartment would significantly and specifically affect the cellular metabolism and accumulation of metals in leaves and seeds. Understanding the role of specific proteins in the distribution of metals inside cells provides opportunities to regulate their transport. This knowledge could be effectively used to optimize cellular metabolism leading to the development of crop plants with better nutritional value that are suitable for a diverse range of environments. Manipulating substrate specificity of transporters, such as NRAMP3 and NRAMP4, could also help breed crop plants that accumulate more beneficial minerals while reducing toxic metal contents in the edible parts.

## Author Contributions

KB wrote the article. KB, SR, TK, MS, and NN discussed and revised the manuscript.

## Conflict of Interest Statement

The authors declare that the research was conducted in the absence of any commercial or financial relationships that could be construed as a potential conflict of interest.
